# Tissue Elasticity Regulated Tumor Gene Expression: Implication for Diagnostic Biomarkers of Primitive Neuroectodermal Tumor

**DOI:** 10.1371/journal.pone.0120336

**Published:** 2015-03-16

**Authors:** Long T. Vu, Vic Keschrumrus, Xi Zhang, Jiang F. Zhong, Qingning Su, Mustafa H. Kabeer, William G. Loudon, Shengwen Calvin Li

**Affiliations:** 1 Neuro-Oncology and Stem Cell Research Laboratory, Center for Neuroscience Research, CHOC Children's Hospital Research Institute, University of California Irvine, 1201 West La Veta Ave., Orange, CA, 92868, United States of America; 2 Department of Neurological Surgery, Saint Joseph Hospital, Orange, CA, 92868, United States of America; 3 Department of Neurological Surgery, University of California Irvine School of Medicine, Orange, CA, 92862, United States of America; 4 Department of Neurology, University of California Irvine School of Medicine, Orange, CA, 92697–4292, United States of America; 5 Department of Biological Science, California State University, Fullerton, CA, 92834, United States of America; 6 Department of Pathology, Keck School of Medicine, University of Southern California, Los Angeles, CA, 90033, United States of America; 7 Department of Pediatric Surgery, CHOC Children's Hospital, 1201 West La Veta Ave., Orange, CA, 92868, United States of America; 8 Bioengineering Research Center, School of Medicine, Shenzhen University, Shenzhen, 518057, Guangdong, China; 9 Department of Surgery, University of California Irvine School of Medicine, 333 City Blvd. West, Suite 700, Orange, CA 92868, United States of America; University of Navarra, SPAIN

## Abstract

**Background:**

The tumor microenvironment consists of both physical and chemical factors. Tissue elasticity is one physical factor contributing to the microenvironment of tumor cells. To test the importance of tissue elasticity in cell culture, primitive neuroectodermal tumor (PNET) stem cells were cultured on soft polyacrylamide (PAA) hydrogel plates that mimics the elasticity of brain tissue compared with PNET on standard polystyrene (PS) plates. We report the molecular profiles of PNET grown on either PAA or PS.

**Methodology/Principal Findings:**

A whole-genome microarray profile of transcriptional expression between the two culture conditions was performed as a way to probe effects of substrate on cell behavior in culture. The results showed more genes downregulated on PAA compared to PS. This led us to propose microRNA (miRNA) silencing as a potential mechanism for downregulation. Bioinformatic analysis predicted a greater number of miRNA binding sites from the 3' UTR of downregulated genes and identified as specific miRNA binding sites that were enriched when cells were grown on PAA—this supports the hypothesis that tissue elasticity plays a role in influencing miRNA expression. Thus, Dicer was examined to determine if miRNA processing was affected by tissue elasticity. Dicer genes were downregulated on PAA and had multiple predicted miRNA binding sites in its 3' UTR that matched the miRNA binding sites found enriched on PAA. Many differentially regulated genes were found to be present on PS but downregulated on PAA were mapped onto intron sequences. This suggests expression of alternative polyadenylation sites within intron regions that provide alternative 3' UTRs and alternative miRNA binding sites. This results in tissue specific transcriptional downregulation of mRNA in humans by miRNA. We propose a mechanism, driven by the physical characteristics of the microenvironment by which downregulation of genes occur. We found that tissue elasticity-mediated cytokines (TGFβ2 and TNFα) signaling affect expression of ECM proteins.

**Conclusions:**

Our results suggest that tissue elasticity plays important roles in miRNA expression, which, in turn, regulate tumor growth or tumorigenicity.

## Introduction

Uncontrolled growth and rapid division of cells characterize cancer. Malignant cancer cells, resistant to programmed cell death, invade surrounding tissue, and possess potential for metastatic migration to other organs. Current cancer treatments (surgery, chemotherapy, radiation) target rapidly dividing cancer cells, resulting in reduction of the tumor size [1], driving the selection of cell subclones with treatment-resistance that leads to recurrence [2]. Such mechanism of cancer cell subclone switching to escape treatment renders malignant cancer incurable. We need to control such dominating subclones for managing cancer progression and posttreatment recurrence by subclonal switchboard signal [3]. However, in some cases, the cancerous cells may reappear and become more resistant to therapy. It is essential to study this cell behavior in a physiologically relevant culture microenvironment.

The treatment-resistance cell subclones are believed to be derived from cancer stem cells (CSCs) [4] and some called cancer as a stem-cell disease [5,6,7]. CSCs reside in a cellular microenvironment (a.k.a., milieu or onco-niche [7], mirror stem-cell niche) where they can maintain their self-renewal characteristics and prevent cell proliferation. For example, glioblastoma-derived CSCs reside in the microvascular niche of brain tumors [8]. CSCs remain stem-cell state until they are out of the onco-niche and this exiting process activates cancer dormant subclones to proliferate. The onco-niche consists of interaction of CSCs with other cells (stromal cells) and the extracellular matrix (ECM) as well as chemical factors (e.g., growth factors). We reported that induced pluripotent stem cells (iPSC) grow along the fiber track in an organotypic brain slice system[9], CSCs form clonal mass [10], and normal neural stem cells migrated toward tumor and differentiated [1] in the native milieu, but not on artificially designed Petri polystyrene (PS) plates. These prompted us to hypothesize that brain environment regulates stem cell behavior. However, a brain environment is a complex of physical and chemical factors, complicating the interpretation of data at the molecular level. Recent publications show that an array of physical metrics plays a vital role for cancer initiation, progression, and metastasis [11]. Intriguingly, a substrate with an elasticity that emulates normal tissue can function as a developmental cue that directs stem cells to differentiate into cells of specific lineages, including mesenchymal stem cells (MSCs) [12] and neural stem cells [13] ([14], page 489). The differences in Tissue-level elasticity of cultural environment were found to determine the fate of MSC cells for their neurogenic, myogenic, and osteogenic differentiations[12]. All of these new reports inspired us to focus on how tissue elasticity regulates gene expression in patient’s derived CSCs. Our results indicate that elasticity-induced gene expression may be regulated by microRNAs in CSCs.

MicroRNAs (miRNAs) are small ~22 nucleotide (nt) non-coding single stranded RNA molecules. About half of miRNA genes are located within introns of coding regions and the other half within intergenic non-coding regions. They are usually transcribed by RNA polymerase II to generate a primary miRNA (pri-miRNA) transcript that is capped and polyadenylated. Primary miRNA (pri-miRNA) can contain multiple stem loop secondary structures that represent precursor miRNA (pre-miRNA), which are cleaved co-transcriptionally within the nucleus by the Microprocessor complex. The Microprocessor complex consists of two gene products bound together, Drosha and DGCR8, which act to attach to cleave primary miRNA. A recent analysis of the genomic location of human miRNA genes suggested that 50% of miRNA genes are located in cancer-associated genomic regions or in fragile sites [15].

The mechanism by which miRNA regulates gene expression is not fully understood. At this time, they are known to play a variety of roles in development and biological processes and have been reported to be important in some cancers. They were initially thought to only silence gene expression via translation repression with the implication that the transcripts are not degraded during repression, but recent studies have determined that miRNAs may primarily silence gene expression by destabilizing and degrading mRNA transcripts leading to reduced protein synthesis. Silencing gene expression by miRNA can be tissue and cell specific.

Tumor microenvironment (stromal cells, soluble factors, ECM) is essential for growth and spread of cancer. Emerging data show that ECM may influence stem cell fate through physical interactions with cells and through transmission of mechanical or other biophysical factors to the cell. Little is known about the physical factors of the microenvironment, including matrix elasticity that may affect gene expression of tumor cells via miRNA. We investigate this possibility with CSCs derived from a primitive neuroectodermal tumor (PNET) obtained from a young patient with an established control cell line of glioblastoma multiforme (GBM) primary cell line (ACBT). Specifically, we studied the global gene expression of CSCs grown on a soft polyacrylamide (PAA) hydrogel plates, used to mimic the elasticity of soft brain tissue, compared to CSCs grown on a standard polystyrene (PS) plates. We propose a novel mechanism, driven by the physical characteristics of the microenvironment by which downregulation of genes occur. We also investigated if tissue elasticity and cytokines affect expression of ECM proteins. Our results suggest that tissue elasticity plays important roles in miRNA expression, which, in turn, regulate tumor growth or tumorigenicity.

## Results

### Asymmetric Expression of Tumor Genes between Tumor Grown on PAA and That on PS

**Fig 1:**
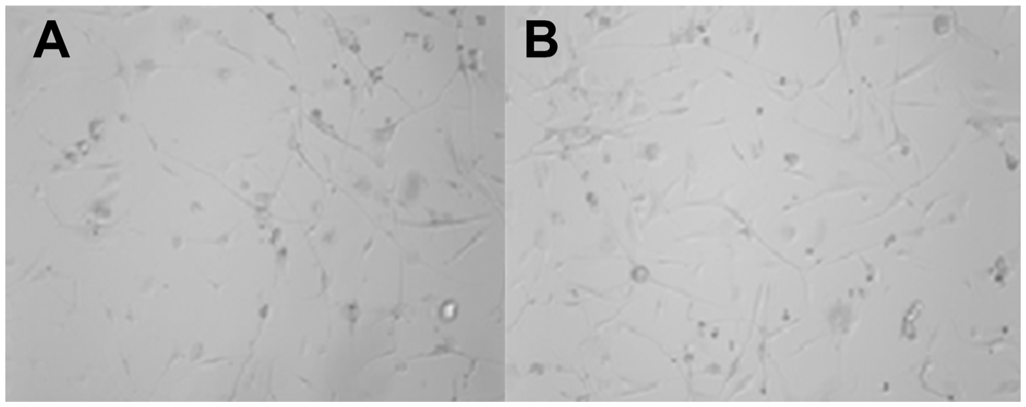
Microscographs of F3Y cells on PS or PAA. Cells were grown on either a standard polystyrene (PS) culture p late (left) or a polyacrylamide hydrogel (right) coated with collagen type I in 5% FBS in Advanced DMEM. (Under 10x magnification).

Primitive neuroectodermal tumor (PNET) cells (F3Y), derived from neurospheres in a neural stem cell (NSC) selection medium, were grown either on standard polystyrene (PS) or on soft polyacrylamide (PAA) hydrogel substrate. The cells retained a similar spindle-shaped fibroblast-like morphology when grown on PS and PAA (Fig. 1). The cells grew slower on PAA than on PS, a phenomenon described previously as micro-environmental mechanical stress-induced apoptosis occurs via the mitochondrial pathway [16]. Six microarray experiments were performed using Affymetrix HG-U133 Plus 2.0 chips, three replicates for each culture condition. The microarray results were normalized by invariant set normalization utilizing DNA-Chip Analyzer (dChip) software [15]. Quantile normalization results were used for comparison purposes for the initial analyses and choice of normalization method.

**Fig 2:**
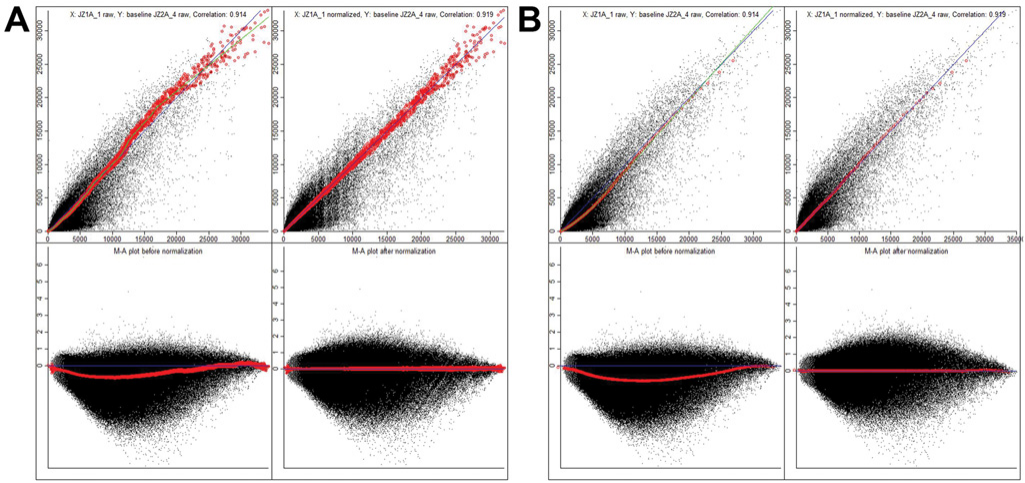
Normalization of gene expression arrays of PNET cells grown on PS or PAA. (A) By ranked invariant set normalization.. All probes were normalized using dChip software implementation of ranked invariant set normalization. The x and y-axis on each of the top panels represent the probes signal intensity values of a PS sample (x-axis) compared to a PAA sample (y-axis) before (left) and after (right) normalization. The bottom graphs are a transformation of the distribution into an MA plot. (Descriptions inside the figure: A: upper left panel—X: JZ1A_1 raw, Y: baseline JZ2A_4 raw, correlation: 0.914; lower left panel—M-A plot before normalization. A: upper right panel—X: JZ1A_1 normalized, Y: baseline JZ2A_4 raw, correlation: 0.919; lower right panel—M-A plot after normalization). **(B)** By quantile normalization. All probes were normalized using dChip software implementation of quantile normalization. The x and y-axis on each of the top panels represent the probes signal intensity values of a PS sample (x-axis) compared to a PAA sample (y-axis) before (left) and after (right) normalization. The bottom graphs are a transformation of the distribution into an MA plot. (Descriptions inside the figure: B: upper left panel—X: JZ1A_1 raw, Y: baseline JZ2A_4 raw, correlation: 0.914; lower left panel—M-A plot before normalization. B: upper right panel—X: JZ1A_1 normalized, Y: baseline JZ2A_4 raw, correlation: 0.919; lower right panel—M-A plot after normalization.)

The presence or absence of a transcript for each gene probed on the array was initially determined using dChip on unnormalized probe intensities for each array. The results demonstrated expression of 57.73% of all genes probed in the PS samples compared to 48.7% of genes probed in the PAA samples (Table 1). All probe intensities of each array were then normalized by either rank invariant set model (Fig. 2A) or quantile normalization model using dChip (Fig. 2B). The scatter plot on the top panels shows the intensity value of a representative probe of a PS sample (PS_1, y-axis) compared to a PAA sample (PAA_4, x-axis) before (top left) and after normalization (top right). The PS and PAA samples plotted were representative samples that displayed differences in gene expression between the two culture conditions. The red points represent probes from the rank invariant set model (Fig. 2A) and from the quantile normalization model (Fig. 2B) used to normalize the arrays. The green line represents the running median normalization curve to which the probes are normalized, while the blue line represents x = y. The greater deviation at high intensities of probes from the rank invariant set indicate that invariant normalization is less reliable for probes with very high signal intensities, while signal intensity ranges that have many invariant probes is more reliable (Fig. 2A).

**Table 1:** Microarray probe summary.

Surface	Median Intensity (unnormalized) ± SD	Presence call % ± SD	
PS	112.33 ± 10.69	57.73 ± 0.35	
PAA	84.67 ± 2.89	48.7 ± 0.7	

Note: The median intensity and the presence call % of all probes on each array was measured using dChip software. The standard deviation (SD) was calculated from three experiments. The presence call % indicates the percentage of all probed gene transcripts that was expressed when cells were grown on the corresponding surface.

MA plots, which are a scaled transformation of the scatter plot of series of thousands of microscopic spots in DNA microarrays, are used to determine if the data needs normalization and test if the normalization worked. Here, we used an MA plot, to compare the signal intensity of genes on PS to gene on PAA where M (y-axis) represents the intensity ratio (log_2_ (control/experimental)) and A (x-axis) represents the average intensity (1/2 * log_2_ (control * experimental)) (Fig. 2, bottom panels). To provide a consistent frame of reference, genes on PS were chosen to be the control and genes on PAA were chosen to be the experimental group throughout this study. The MA plot before (Fig. 2A, bottom left) and after normalization (Fig. 2A, bottom right) demonstrated that, the change in gene expression was not evenly distributed between positive and negative expression (Fig. 2A and B). The data shows more points below an M value of zero (x-axis) than above an M value of zero, which indicates that, more genes downregulated in cells grown on PAA than in cells on PS.

**Fig 3:**
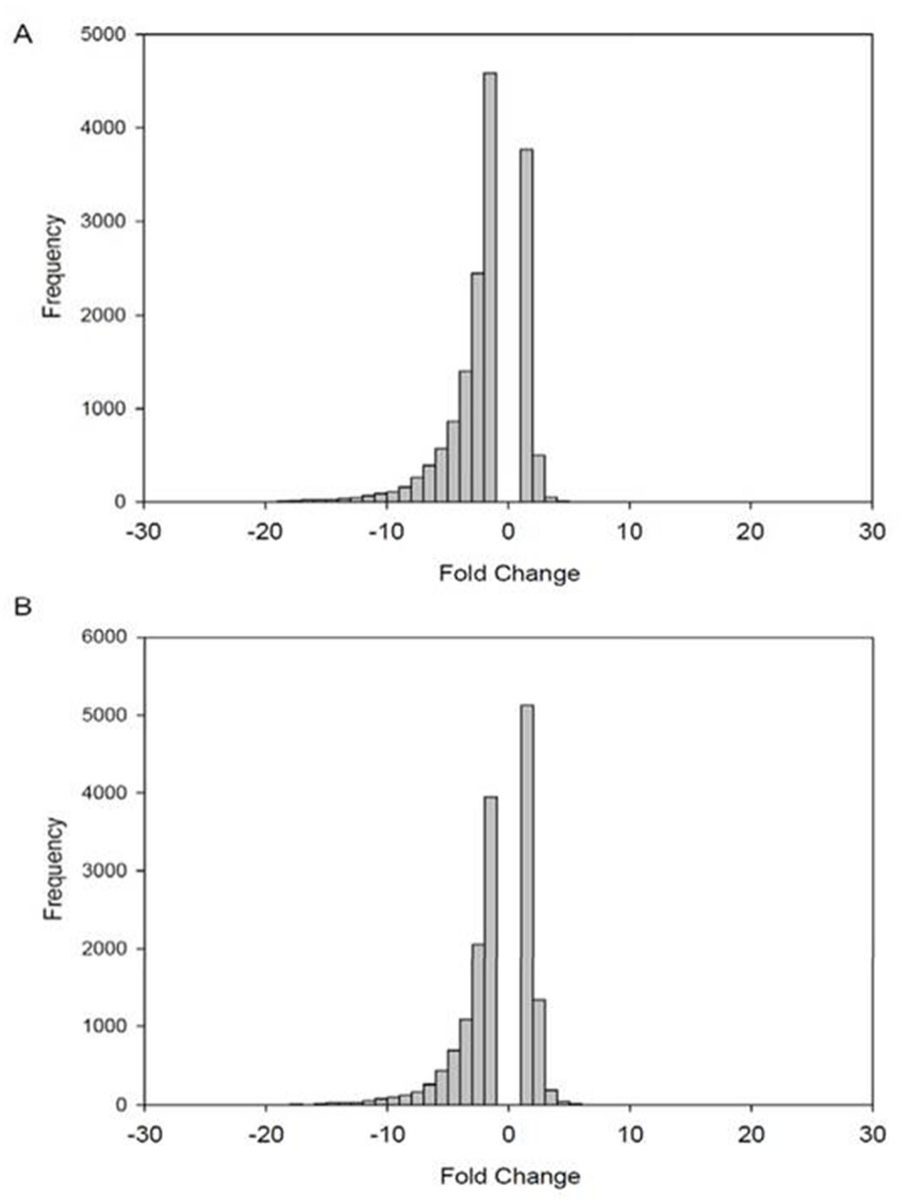
Choice of normalization method affects gene expression balance. Histograms showing the distribution of up or downregulated genes normalized by (A) invariant set normalization or (B) Quantile normalization grown on PAA compared to PS. A negative fold change (FC) represents genes downregulated on PAA and a positive FC represents genes upregulated on PAA.

**Fig 4:**
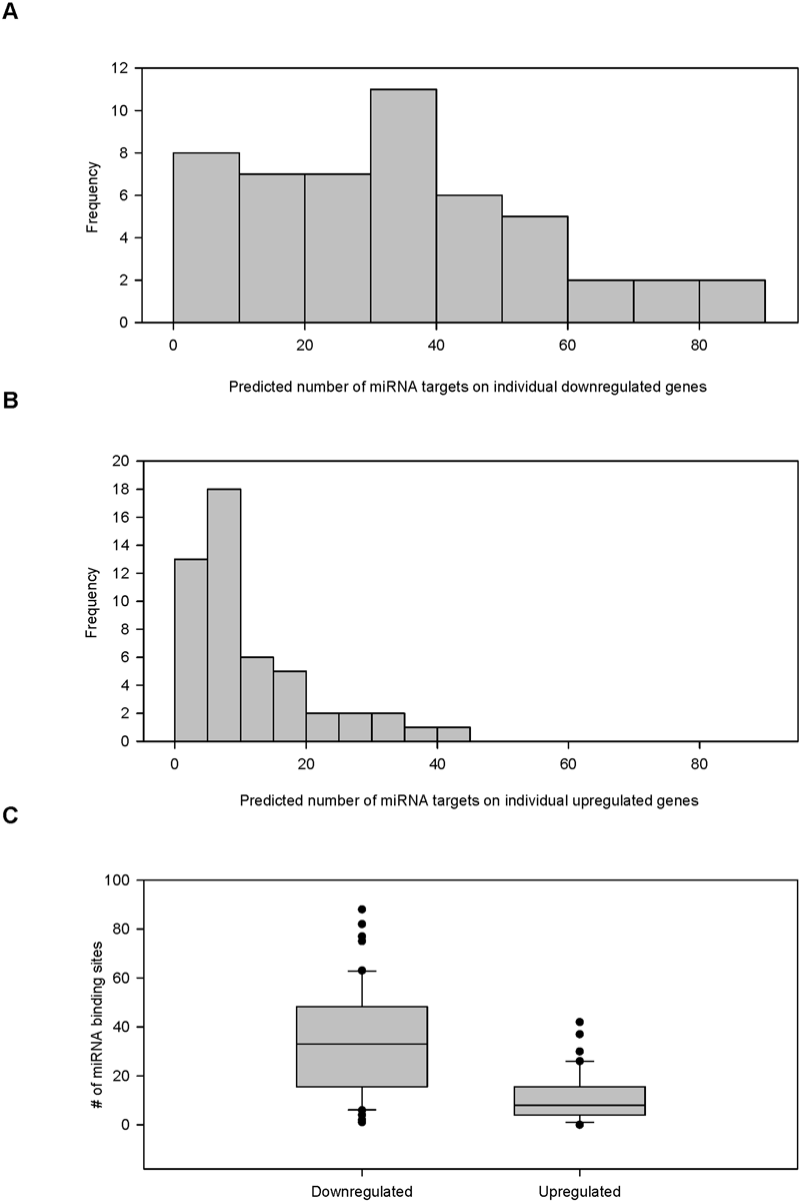
Greater number of miRNA binding sites predicted from probes downregulated on PAA. A random samples of 50 probes upregulated on PAA (FC > 2) was compared to 50 probes highly downregulated on PAA (FC < -10). MicroRNA target sites were predicted from the 3' UTR of each probe for each set collected using the microRNA.org resource. Histograms of the predicted number of miRNA targets are for the probes (A) downregulated on PAA and (B) upregulated on PAA. (C) Box p lot that compared the distribution of the two sets of probes.

To confirm the above finding, a histogram of the fold change of genes between the PS and PAA conditions, normalized by rank invariant set normalization (Fig. 3A) or quantile normalization (Fig. 3B), was used to express that genes were skewed towards downregulation on PAA. This was indicated by the greater amount of genes that have a negative fold change compared to the amount of genes that had a positive fold change from PS to PAA. The effect of asymmetric gene expression was greater in the normalization method of rank invariant set and dampened in quantile normalization (Figs. 2, 3, and 4). This effect was more difficult to observe in Fig. 2 because the high density of the points in the MA plots masks the distribution of the probes. Since the quantile normalization process assumes that the upregulated and downregulated genes are evenly distributed, the quantile normalization results were invalidated. We therefore relied upon the normalization results from rank invariant set for subsequent data analyses. Finally, to identify differentially regulated and unregulated genes, the two conditions (PA and PAA) were compared and filtered to include only the genes that met the following well-established criteria: (1) probes with a *p*-value less than 0.05 using a paired t-test. (2) Signal intensity is greater than 100 in 50% of the probes. (3) Signal intensity is with a lower than 90% confidence bound of fold change (FC).As such, 15,515 probes were filtered from the original 54,639 probes of the gene chip. Following filtration, the distribution of differential gene expression between PS to PAA was skewed towards a negative fold change. Of the remaining 15,515 filtered probes, 1,220 (7.86%) were unregulated (|FC| < 1.2), 7,139 (46.01%) were found to be differentially regulated (|FC| > 2), 575 (3.70%) were upregulated (FC > 2) when grown on PAA compared to PS, and 6,564 (42.30%) were downregulated (FC < -2) when grown on PAA compared to PS (Table 2). At greater fold changes, there was an increase in the ratio of downregulated probes to upregulated probes (Fig. 3, Table 2).

**Table 2:** Proportion of probes up or downregulated on PAA.

Fold Change (FC)	Total Probes	Downregulated Probes (negative FC)	Upregulated Probes (positive FC)
|FC| < 1.2	1220	603	617
|FC| < 2	8351	4589	3762
|FC| > 2	7139	6564	575
|FC| > 3	4201	4131	70
|FC| > 5	1874	1871	3
|FC| > 10	378	378	0

Note: Proportion of probes up or downregulated on PAA. As an example, 7139 probes had a |FC| > 2 and of those, 6564 were downregulated and 575 were upregulated.

Thus, all the above aggregate findings (unnormalized, normalized, MA plot, histogram, and filtered comparisons) consistently led to the conclusion that the substrate was likely a factor in the down regulation of gene transcripts in tumor cells on PAA compared to cells on PS.

### Downregulation of Genes on PAA by miRNA

The high degree of genes downregulated on soft polyacrylamide (PAA) hydrogel suggests a mechanism that preferentially downregulated messenger RNA (mRNA). Mechanisms such as DNA methylation and histone modification can downregulate mRNA expression, but they can also upregulate its expression depending on the default state of the gene. Downregulation of gene expression leads to a reduction in production of gene products and, ultimately, to a decrease of effector activity. However, reductions in the activity of the gene products could have the same effect of decreased effector activity. Regulation by microRNA (miRNA) is a post-transcriptional process that silences mRNA expression by binding of the miRNA to complimentary regions on the three prime untranslated regions (3' UTR) of mRNA ultimately causing both the inhibition of translation and the destabilization and degradation of mRNA. Thus, we wanted to test whether miRNA silencing was more closely associated with genes that were downregulated than for those upregulated on PAA.

To confirm this close association, we took a random sample of 50 probed genes that were highly downregulated on PAA as evidenced by a fold change of less than-10 (FC < -10) and compared them to a random sample of 50 probed genes that were upregulated on PAA as indicated by a fold change greater than 2 (FC > 2). Our analysis found a statistically significant difference in gene regulation between genes highly downregulated and upregulated on PAA.

A web resource (www.microRNA.org) was used to collect miRNA target sites of downregulated genes that had a mirSVR algorithm score (a measure of probability that a particular miRNA would repress a gene) of less than-0.10, an established parameter. The probability of miRNA binding to a site and the resulting degree of repression was with mirSRV scores lower than-0.10. Conversely, the probability of repression sharply drops at mirSRV scores greater than-0.10. A more stringent criterion may miss possible miRNA binding sites. The number of predicted miRNA binding sites for each downregulated gene in the two sample sets (PS versus PAA) was collected (S2 Dataset) and histograms of the predicted number of miRNA targets for the probes downregulated on PAA (Fig. 4A) and upregulated on PAA (Fig. 4B) were created along with a box plot that compared their relationship (Fig. 4C).The result shows the number of predicted miRNA bindings sites within genes downregulated on PAA was greater than in genes upregulated on PAA (Fig. 4A, B, and C). The box plot and subsequent box plots are represented by the median and the 10th, 25th, 50th, and 90th percentiles with circles representing outliers. The number of predicted miRNA binding sites that were downregulated on PAA (mean = 33.460, SD = 21.647) compared to the predicted miRNA binding sites upregulated on PAA (mean = 10.840, SD = 9.778) was statistically significant (*p* < 0.001). This supports the idea that many transcripts were downregulated on PAA via miRNA silencing.

**Fig 5:**
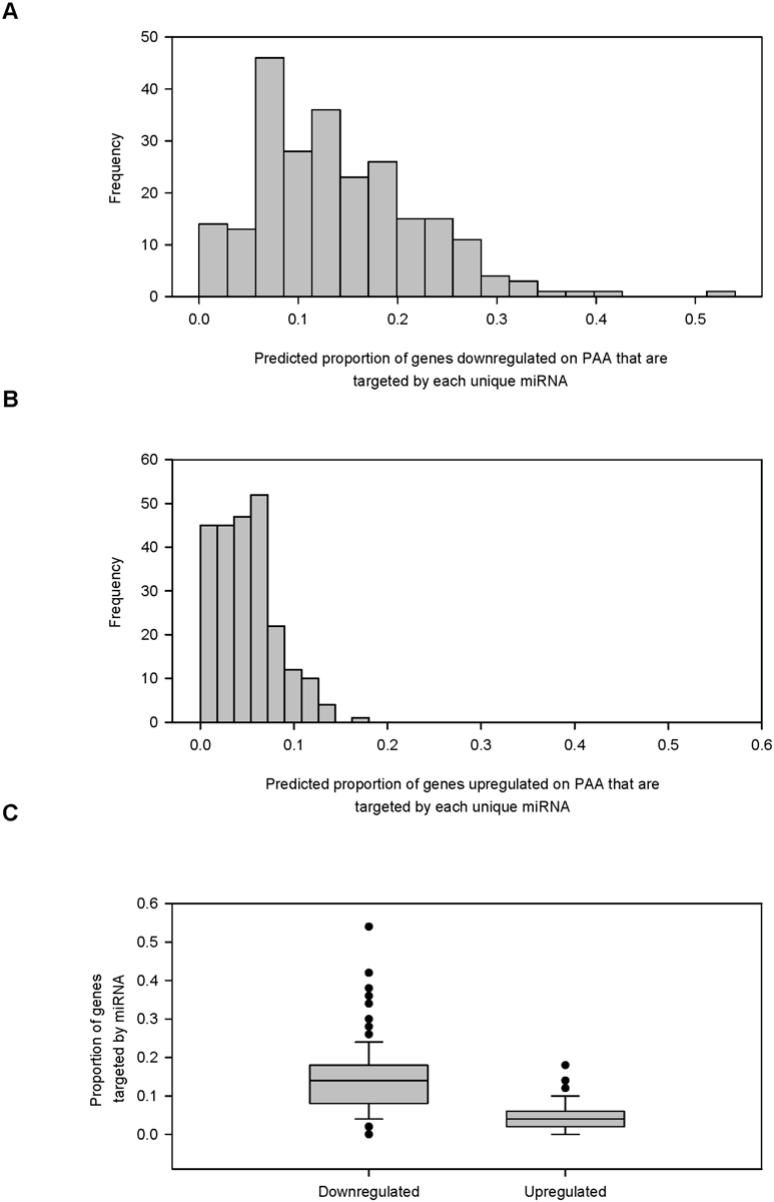
Downregulation of genes on PAA by specific miRNAs predicted. A random samples of 50 p robes upregulated on PAA (FC > 2) was compared to 50 probes highly downregulated on PAA (FC < -10). MicroRNA target sites were predicted from the 3' UTR o f each probe for each set using the microRNA.org. Histograms of the proportion of genes predicted to be targeted by each miRNA was created for the set of genes (A) downregulated and (B) upregulated on PAA. (C) Box plot that compared the distribution of the two sets of probes.

The proportion of individual genes within each set that were targeted by miRNA was compared between the downregulated and upregulated sets and demonstrated that the downregulation of mRNA was likely caused by an increase of individual miRNA levels, which was influenced by the elasticity of the surface substrate (Fig. 5). Again, miRNAs were predicted to bind a greater proportion of genes downregulated on PAA than those upregulated on PAA. The Wilcoxon signed-rank test showed that the proportion of predicted genes targeted by individual miRNA was greater in genes downregulated on PAA than upregulated on PAA (Z = -12.085, *p* <. 001). This shows that the downregulation of transcripts could be caused by the increase in specific miRNAs on PAA.

**Fig 6:**
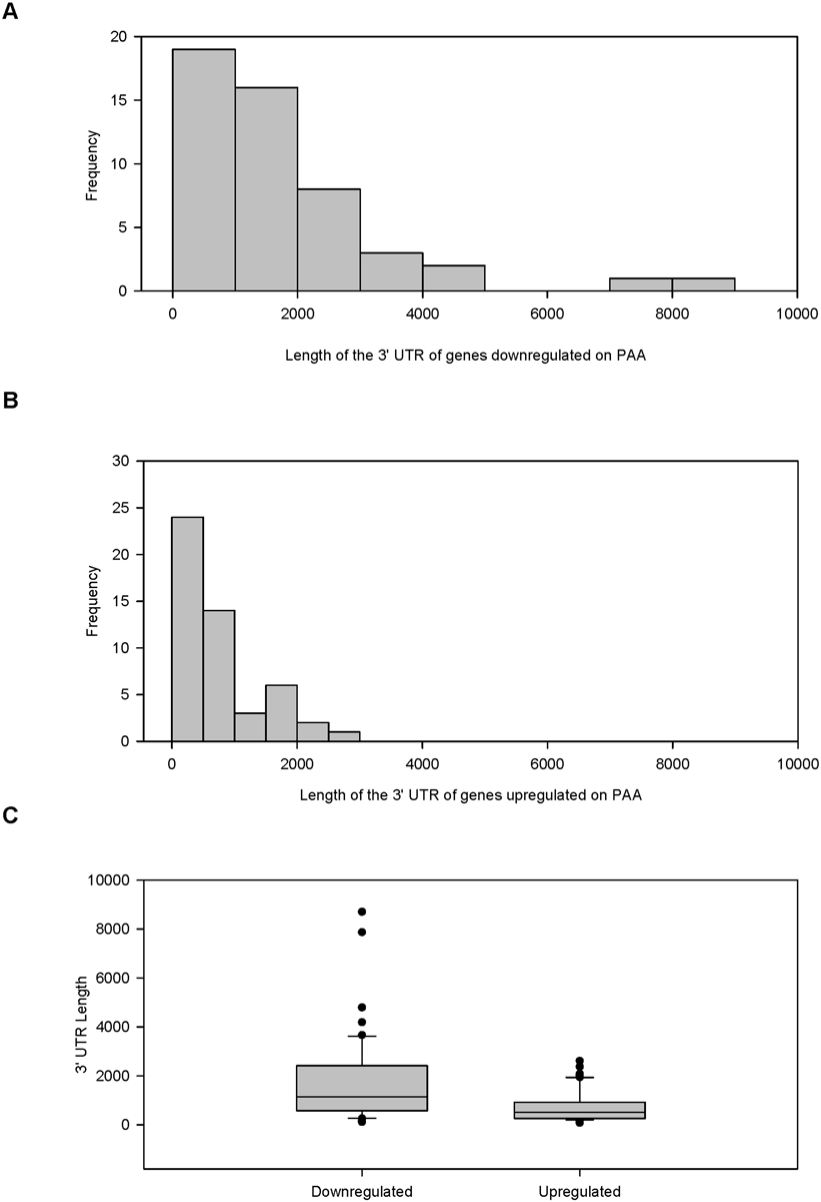
Greater proportion of probes downregulated on PAA have a longer 3' UTR. A random samples of 50 probes upregulated on PAA (FC > 2) was compared to 50 probes highly downregulated on PAA (FC < -10). MicroRNA target sites were predicted from the 3' UTR of each probe for each set using the microRNA.org resource using a conserved mirSVR scores of < -0.1 as a filter to collect predicted miRNA target sites. Histograms of the length of the 3' UTR for the probes (A) downregulated on PAA and (B) upregulated on PAA. (C) Box plot that compared the distribution of the two sets of probes.

During the process of collecting miRNA binding sites from the two sets of differentially regulated genes, we also recorded the length of the 3' UTR for each probe. The 3' UTR contains the miRNA binding sites and a longer 3' UTR should correlate to more potential miRNA binding sites. The length of the 3' UTR for each probe downregulated on PAA was compared to the probes upregulated on PAA (Fig. 6). The Wilcoxon signed-rank test shows that probes downregulated on PAA have longer 3' UTRs than the probes upregulated on PAA (Z = -3.837, *p* <. 001).

Thus, microenvironment (elasticity) may lead miRNA functional changes, which in turn moderate mRNA production. It appears that gene regulation is consistently different on the PS substrate compared to that on the PAA substrate. We examined what specific miRNA species may change these substrate-regulated genes.

### Identification of Tissue Specific miRNA Candidates

If miRNAs that bind in different proportion to genes downregulated on PAA could be identified, they may be candidates for brain specific gene regulation and be the agents of selective repression by the miRNA. The miRNA prediction relies on identifying sequences on the 3' UTR that can correspond to several different miRNAs. Prediction of miRNA is determined, in part, by the identity of the mature miRNA sequence and its seed region that consists of the initial 2–7 base pairs on the 5' end of the mature miRNA that binds to the 3' UTR. The collection of miRNAs predicted was annotated with its chromosomal location(s), mature miRNA sequence, and mirBase accession number for comparison purposes (S2 Dataset). Of particular interest are predicted miRNAs within the miRNA clusters of C14MC and C19MC. The C14MC cluster (also named miR-379–410 cluster) is located on the chromosomal locus 14q32.31; several miRNAs within the cluster were predicted from genes downregulated on PAA. C14MC, a maternally imprinted cluster, has miRNAs that are processed from a long non-coding RNA containing dozens of intronic miRNA, which has tissue specific expression that is the strongest in the brain tissue of monkeys [17]. Other interesting miRNAs that were enriched on PAA were predicted within the C19MC cluster located on the chromosomal locus 19q13.42, the MIR371–373 cluster, and the MIR302–367 cluster located on the chromosomal locus 4q25 (S1 Dataset). All three clusters contain a similar miRNA sequences that characterize stem cells, miR-520, miR-302, miR-372, and miR-373 and have an identical seed sequence of AAGUGC (S1 Dataset). The identical seed sequences of these miRNA demonstrate that the prediction of miRNA can be non-specific to a single miRNA. The C19MC cluster also contains intronic miRNA that is processed from a large non-coding RNA. The amplification of miRNAs within the C19MC cluster is associated with central nervous system primitive neuroectodermal embryogenic tumor (CNS-PNET), which matches the type of tumor under study.

**Fig 7:**
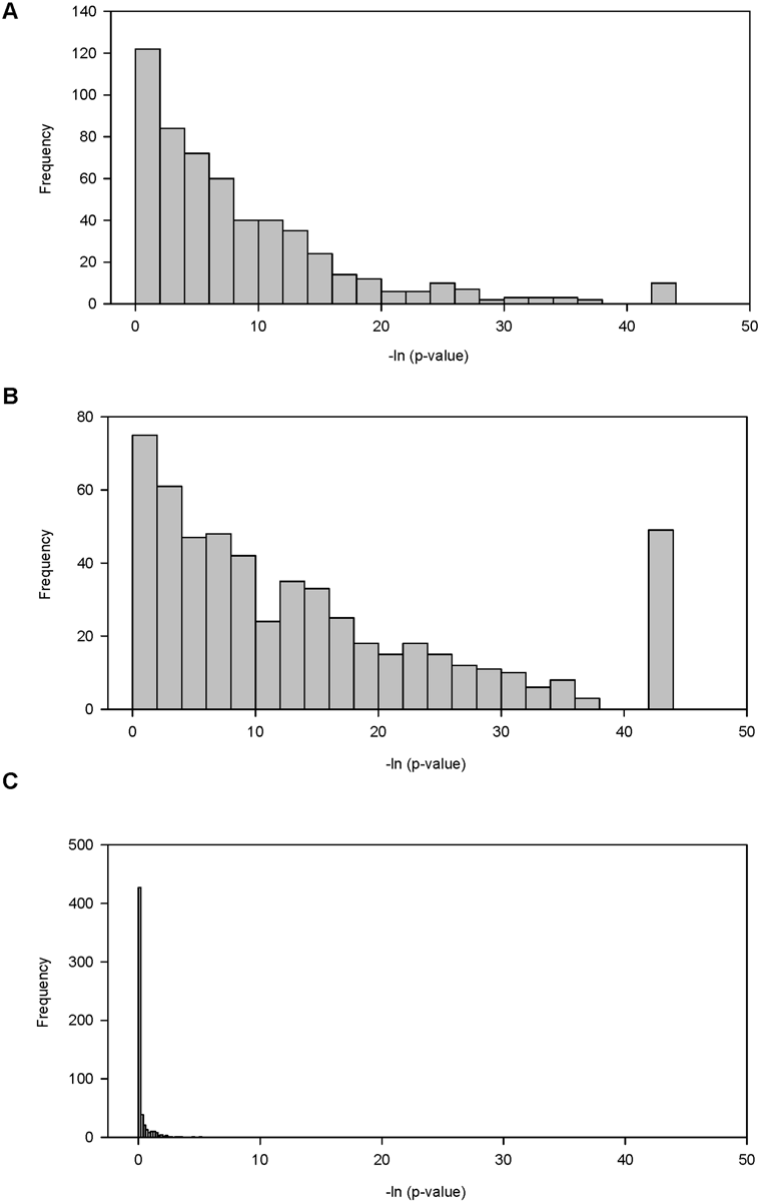
MicroRNAs predicted from sets of genes downregulated on PAA were significant different from genes unregulated on PAA. MicroRNA targets from genes sets that were, downregulated (subset of genes FC < -2), highly downregulated (FC < -10), or upregulated (FC > 2) were compared to predicted miRNA targets in the set of genes that was unregulated (FC < 1.2) utilizing Diana-mirExtra to identify miRNA that were statistically significant (p < 0.5) between the sets. The histograms show the number of predicted miRNAs that were statistically significant when comparing the set of unregulated genes to a set of genes (A) highly downregulated, (B) downregulated, or (C) upregulated. The lowest p-value calculated by Diana-mirExtra was 1 x 10–19 which corresponds to the peaks at 43.74.

Although we were able to predict possible miRNA that bind to target sites, we lacked a statistical test to show that the predicted miRNAs were statistically significant. To address this, we used DIANA-mirExTra (www.microrna.gr/mirextra), a web server that uses an alternative method to detect and score miRNA target binding sites using expression data. Diana MicroT v3.0 web server Probe sets (Table 2) were used to compare differentially regulated genes sets to a group of unregulated genes (|FC| < 1.2) for prediction miRNAs that were differentially regulated when grown on PAA. Histograms were then created to show the proportion of predicted miRNAs that were significantly different from the unregulated gene set (Fig. 7). The results demonstrate that the genes highly downregulated (FC < -10) and downregulated (FC < -2) on PAA (Fig. 7A,B) had a significantly larger proportion of predicted miRNA (*p* < 0.05) than the genes upregulated (subset of 500 probes with a FC > 2) on PAA (Fig. 9C). Only 0.9% (5/555) of the predicted miRNA within the upregulated genes set was statistically greater from the unregulated gene set (Fig. 7C, S2 Dataset), while 70.8% (393/555) of the miRNA predicted within the highly downregulated genes was predicted to be greater than the unregulated set (Fig. 7A, S2 Dataset). Interestingly, the subset of 500 probes with a FC < -2 had 80.7% of the miRNA significantly greater than the unregulated set (Fig. 7B, S2 Dataset), which was an unexpectedly higher proportion than FC < -10 set. Subsets were used because of technical limitation of the web server that prevented the processing of very large data sets such as the set of genes with a FC < -2 (6,564 probes) or FC < -5 (1,871 probes). The lowest *p*-value calculated by Diana-mirExtra was 1 x 10^–19^ which corresponds to the peaks at a-ln (*p*-value) of 43.74 (Fig. 7A and B).

### Functional Gene Analysis

**Fig 8:**
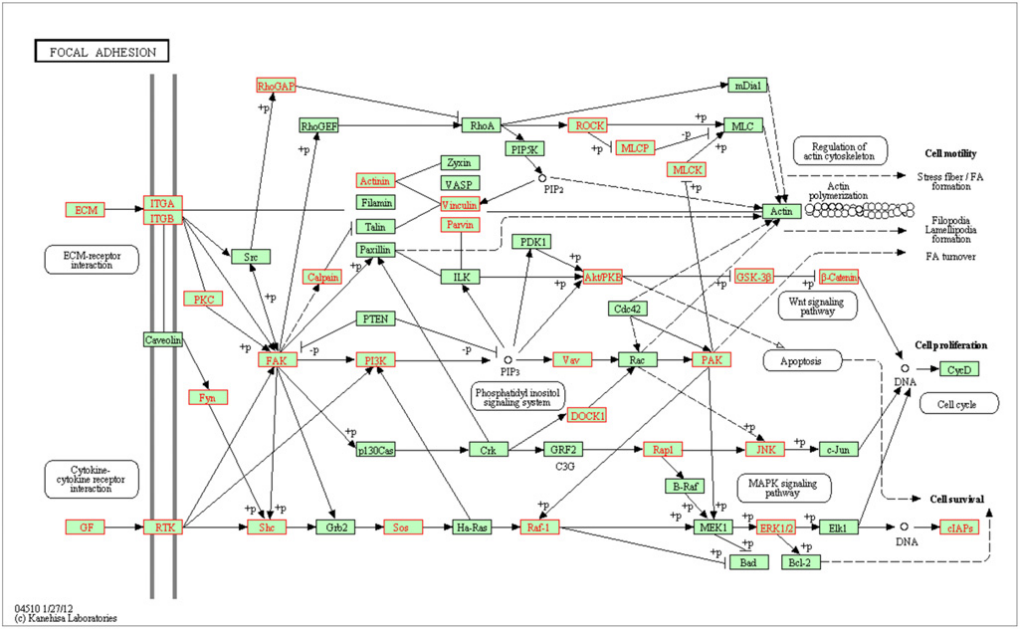
Functional gene enrichment of the KEGG focal adhesion pathway. Differentially regulated genes were mapped onto a KEGG pathway (hsa04510) that was found to be enriched between the culture conditions. Genes highlighted red indicates probes downregulated on PAA (FC < -2), including ITGA, ITGB, PKC, Fyn, RhoGAP, FAK, PI3K, Calpain, Actinin, Vinculin, Parvin, Vav, DOCK1, Rap1, JNK, ERK1/2, GSK-3β, β-Catenin, MLCP, ROCK, RTK, Shc, Sos, Raf1. No probes highlighted were upregulated on PAA (FC > 2) within this pathway.

Gene Ontology (GO) terms are defined by three attributes of wild-type gene products: their molecular function, the biological processes in which they play a role, and their subcellular location [18]. To search for functional gene enrichment between the two conditions, the list of differentially regulated genes with |FC| > 2 was used to search for enriched annotated Gene Ontology (GO) terms, Kyoto Encyclopedia of Genes, and Genomes (KEGG) pathway terms using The Database for Annotation, Visualization, and Integrated Discovery (DAVID) software. DAVID provides biological meaning behind large list of genes and KEGG is a collection of online databases dealing with genomes, enzymatic pathways, and biological chemicals. The KEGG PATHWAY database records networks of molecular interactions in the cells and variants of those networks specific to particular organisms. In each database, genes are annotated with a list of terms describing its functions. The list of terms from differentially regulated genes was compared to a list taken from background genes and a probability that a particular function is enriched in the differentially regulated set compared to the background list is made. The list of differentially regulated genes consists of 7,139 probes representing approximately 30% of the genes expressed. From this comparison, we observed a large number of terms that were enriched in the list of differentially regulated genes that also represented a broad collection of GO terms and KEGG pathways (S3 Dataset). A relevant KEGG pathway that was shown to be enriched was the focal adhesion pathway (hsa04510) which mediates cell anchorage to the ECM and can sense and react to changing cell elasticity. The differentially regulated genes were mapped onto the focal adhesion pathway (Fig. 8); genes highlighted in red indicate probes differentially regulated on PAA (|FC| > 2). Inspection of these genes revealed that all of the probes were downregulated on PAA instead of being upregulated. Interestingly, this bias was also seen on most, but not all, terms and pathways that were found to be functionally enriched on PAA, suggesting that microenvironment (tissue elasticity) play a key role for gene downregulation.

**Fig 9:**
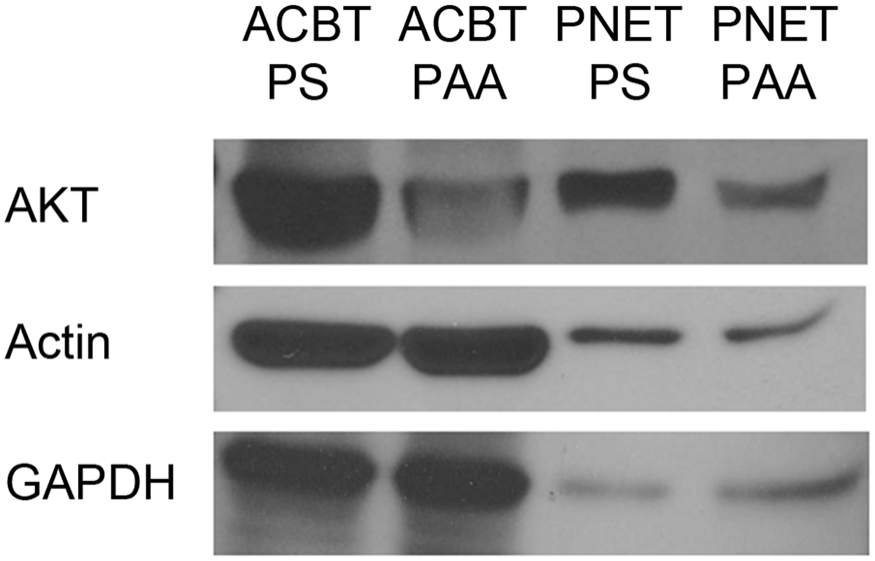
Western blot of ACBT and F3Y cell lysate for AKT on PS or PAA. GAPDH and Actin served as loading controls.

One of the genes regulated in the focal adhesion pathway related to matrix interaction is AKT, an oncogene that plays a variety of roles including anchorage-independent cell growth, apoptosis, metabolism, and angiogenesis. To observe if the downregulation of AKT gene transcripts resulted in lower protein expression and to show if the surface elasticity affected other cells, western blotting was performed on PNET (F3Y) cells and glioblastoma (ACBT) cells grown on PS or PAA. We observed that both the PNET and the glioblastoma cells had lower AKT gene products on PAA compared to PS (Fig. 9), suggesting that these two cell lines may share regulation pathways under these conditions.

The GO terms that are enriched can help identify multiple types of gene regulation depending on growth in a softer culture environment. Such a regulation may lead to an increase in miRNA production in the cell. The large quantity of terms enriched and their broad range made it difficult to separate the terms that may have played a role in the upregulation of miRNA on PAA from the terms that are an effect of the increased miRNA production. To separate the terms that regulate these processes, we have to interpret the terms enriched using prior knowledge of the biology of these processes and design experiments to confirm them. Though not apparent at first, terms enriched in the differentially regulated gene set included RNA binding, transcription, and spliceosome that are important in the mechanism of alternative polyadenylation and alternative splicing. We propose alternative polyadenylation in this study to have a role in the regulation of miRNA production.

### Downregulation of miRNA Processing Genes

**Fig 10:**
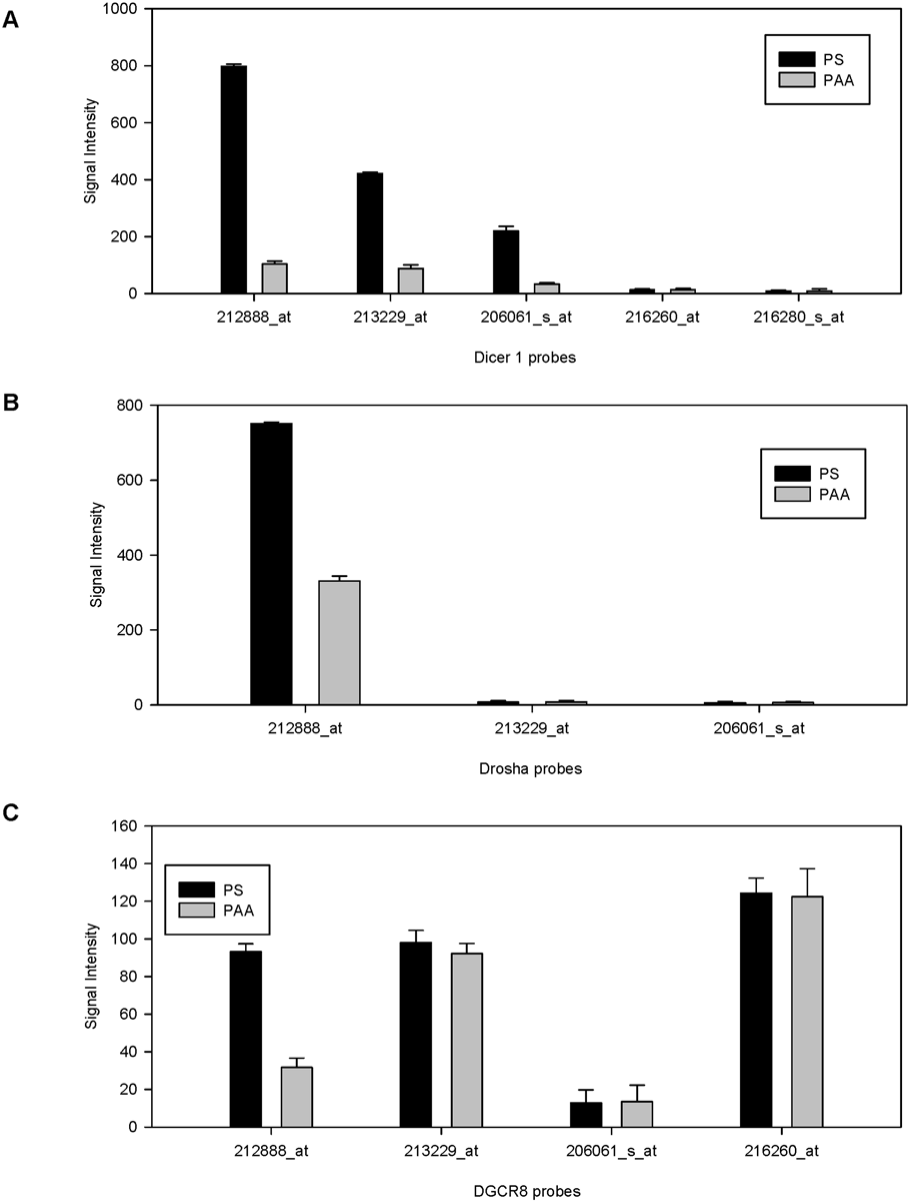
Downregulation of miRNA processing genes on PAA. Microarray signal intensities of miRNA processing enzymes were compared on PS and PAA. Error bars represent standard deviation.

To evaluate our alternative hypothesis regarding polyadenylation and its regulation of miRNA production, we examined proteins that process miRNA. The Drosha and Dicer genes were both noted to have been down regulated on PAA (Fig. 10A and B). DGCR8, an RNA binding protein, assists the RNase III enzyme Drosha in the processing of miRNAs [19]. However, when examined, DGCR8 did not show significant downregulation on PAA (Fig. 10C). One of its probes was downregulated but because the probes were at low signal intensities (~100), the background signal noise, and possible systematic errors were assumed to play a larger relative role. Dicer had been previously found to be involved in a negative feedback loop with a miRNA, let-7; an increase of let-7, processed by Dicer to generate mature miRNA, also silences Dicer.

The Dicer, Drosha, and DGCR8 genes were examined, using www.microRNA.org to search their 3' UTR regions, to predict conserved miRNA binding sites that were below a mirSVR threshold score of-0.10. From this criterion, Dicer had 57 miRNAs that were predicted to bind to its 3' UTR compared to 25 for Drosha and 13 for DGCR8. The list was compared to the predicted miRNAs previously found significant in the set of genes highly downregulated (FC < -10) on PAA compared to the unregulated gene set (Fig. 7A, S2 Dataset). Of the 57 miRNA binding sites identified on Dicer, 47 of those were predicted to be downregulated on PAA (Table 3).

**Table 3:** Negative feedback regulation of miRNA processing enzymes by miRNA.

mRNA	Dicer	Drosha	DGCR8
Total conserved miRNAs predicted to bind to each mRNA (microRNA.org)		25	13
Number of conserved miRNAs found significant (Diana-mirExTra)		24	13
**mRNA**	**Dicer**	**Drosha**	**DGCR8**
Total miRNAs predicted to bind to each mRNA (Diane-microT v3.0)		0	4
Number of miRNAs found significant (Diana-mirExtra)		0	4

Note: The number of miRNA that bind to each mRNA was predicted using either microRNA.org using a threshold score of less than-0.10 or Diana-microT v3.0 with a threshold score of greater than 7.3. Diana-mirExtra was used to identify the miRNAs that were significantly downregulated on PAA (FC < -10, p< 0.05) from unregulated genes and used here to determine the proportion of predicted miRNA significant compared to the total miRNAs predicted for each miRNA processing gene.

An alternative miRNA prediction algorithm, Diana-microT v3.0 (miTG score > 7.3), was used to identify 54 predicted miRNAs binding sites for Dicer with 46 found to be significant from Diana-mirExtra (Table 3). Significant miRNAs were also found for Drosha and DGCR8 using the same methods. This is evidence that groups of miRNAs expressed together have a role in the negative feedback of miRNA production and builds on previous studies of a single miRNA, let-7 and Dicer negative feedback.

### Identification of Transcripts Containing Intron Sequences for Cells Grown on PAA

Among the differentially regulated probes sets, there were genes that contained both upregulated and downregulated probes which may indicate they are isoforms of the genes that were regulated by the tissue elasticity of the surface medium. As many probes exhibit this behavior, we first examined epidermal growth factor receptor (EGFR). The EGFR probe was highly down regulated on PAA. EGFR is an oncogene that is overexpressed in many cancers including 40–50% of glioblastomas and epithelial cancers. EGFR is one of the receptor tyrosine kinases enriched in the focal adhesion pathway analysis.

EGFR is a cell surface receptor that interacts with ECM proteins, some of which were highly downregulated on PAA. We mapped the EGFR genes using Ensembl and added the HGU133 Plus 2.0 probe tracks to align the probes onto the gene sequence to locate possible splice variants that were regulated by tissue elasticity. Unexpectedly, two of the probes (232541_at and 232925_at) were mapped to intron sequences for EGFR. It had an expression signal that indicated the presence of the transcripts on PS but its downregulation on PAA. Mature mRNA have intron sequences spliced out of the transcript and the identification of introns in EGFR transcripts point to an alternative isoform containing intron sequences. Other ECM genes were next checked for the existence of introns within their transcript and we found more evidence of introns within these transcripts. Though not every single gene examined contained intron sequences, it was common within probes that were highly downregulated on PAA. The presence of introns within transcripts may be caused by the softer surface environment of PAA, which reflect a process that may also occur in native cells. In addition, many genes only have a few probes within introns, which was a limitation of the array platform. We then examined a non-coding RNA, maternally expressed gene 3 (MEG3). MEG3 is a well-studied gene that has many highly downregulated probes when grown on PS compared to PAA. It is neighboring the C14MC cluster that we identified earlier and may be a source of miRNA that was upregulated on PAA. Most of the probes of MEG3 reside in intron regions and all of them were highly differentially regulated on PAA.

### Characterization of PNET Invasiveness Induced by Cytokines on PAA

We emphasized here that we used high-throughput gene expression experiments to identify or predict the role of genes involved in biological conditions of interest like stiffness, a scheme that has been widely applied. Keklikoglou and colleagues show that overexpression of miR-520/373 members reveals a strong downregulation of transforming growth factor-β (TGF-_β_) signaling and a negative correlation between miR-520c and TGFBR2 expression was observed in estrogen receptor negative (ER(-)) breast cancer patients[20]. Liu and Wilson made a direct connection that miR-520c and miR-373 increased the expression and activity of MMP9 in 3D type I collagen gels [21]. TGFβ and TNFα regulate the expression and activation of MMP9 [22] while IL-1 regulates MMP9 activity[23]. Taken together, we rationalized we could measure the cytokine regulated MMP9 activity to proposal an involvement of miRNA expression.

**Fig 11:**
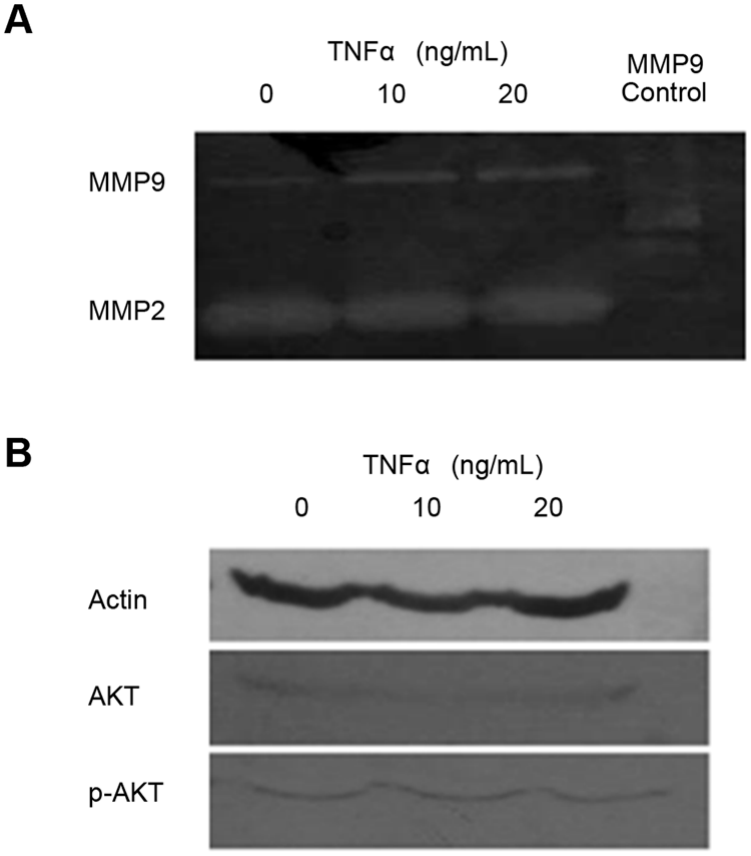
Gelatin zymography and Western blot analysis of F3Y cells induced by TNFα. 0, 10, or 20 ng/mL TNF was added to each culture. Purified MMP9 protein was used the MMP9 control. (A) Gelatin zymography was performed on the conditioned medium and (B) western blot was performed on the cell lysates.

**Fig 12:**
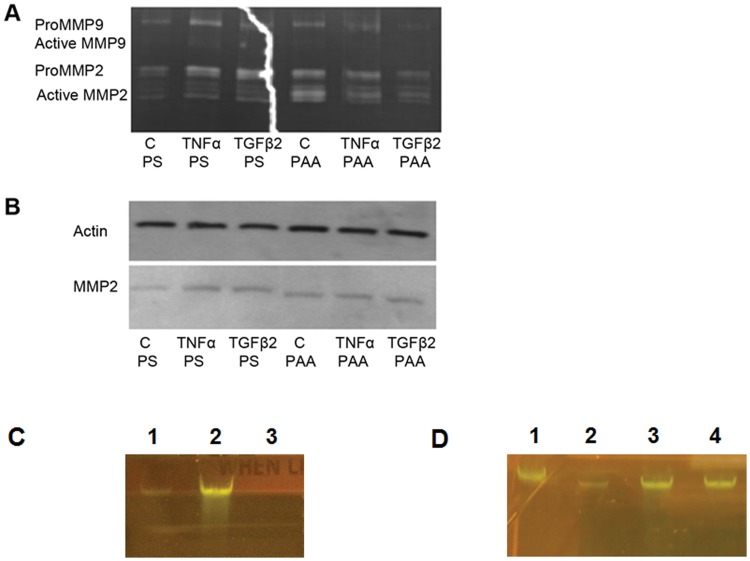
Tissue elasticity modifies F3Y cells physiology and regulation of MMP activity via miRNA regulated signaling pathway. F3Y cells were grown on PS or PAA without cytokines (C, Control), 10 ng/mL of TNFα, or 10 ng/mL of TGFβ added. (A) Sepharose bead pull down of cell conditioned medium followed by gelatin zymography. (B) Western blot analysis of the F3Y cell lysates for MMP2. (C) Agarose gel electrophoresis of PCR products, Lane 1: mir-520c pri-miRNA expression on PS; Lane 2: mir-520c pri-miRNA expression on PAA; Lane 3: No template control. (D) Agarose gel electrophoresis of PCR products, Lane 1: CD44 expression on PS; Lane 2: CD44 expression on PAA, Lane 3: CD44 expression on PS + acrylamide monomer solution; Lane 4: CD44 expression on PS + supernatant from PAA plate (PAA degradation products).

MMPs are secreted by cells to degrade ECM, to remodel and maintain their microenvironment. Migratory cells secrete MMPs to break down the basement membrane to pass through the tissue barrier. In stem cells and cancer cells, MMP2 and MMP9 are regulated by cytokines such as TGFβ2 and TNFα. In our experiments, we added 0, 10, 20 ng/mL TNFα to F3Y cells grown on PS and assessed their enzymatic activities using gelatin zymography and Western blotting. The results show that MMP2 was slightly induced by 10 ng/mL of TNFα and greater levels of the cytokine show no additional effect (Fig. 11A). AKT and p-AKT are proteins downstream from TNFα signaling and were used to determine if the phosphorylated AKT (p-AKT) was affected but no change was observed (Fig. 11B). Similar experiments were also done on PS and PAA conditions with the addition of either zero (C, control) or 10 ng/mL of TGFβ2 or TNFα (Fig. 12). To concentrate the amount of MMP loaded onto the well, Gelatin Sepharose 4Bbeads were used to bind to MMP proteins. On PS, both TNFα and TGFβ2 were able to induce MMP9 and MMP2 activity in the condition medium of F3Y cells from the control (Fig. 12A). On PAA, the reverse occurred when either TNFα or TGFβ was added on cells grown on PAA where there was a repression of MMP activity (Fig. 12A).Western blotting of MMP2 showed nearly equal levels of MMP2 protein (Fig. 12B). Deb and colleagues show that the lower molecular weight, gelatin-degrading activity is an activated form of MMP-2 in U87 human glioma cells [24]. Han and colleagues show that multiple isoforms of MMP9 are induced with TGFβ or TNFα [22]. In Fig. 12A, we observed multiple bands, indicating that multiple forms of MMP2 were induced. These might include pre-MMP2 (72 kDa) and active MMP2 (62 kDa). However, certain conditions led to generate the 65 kDa and 68 kDa MMP-2 isoforms [25]. We didn not know why in Fig. 12B, the MMP2 molecular weight in PAA-cultured cells seemed to be a little smaller than those in PS-cultured cells as indicated by Western blot analysis. These might be result from alternative splicing specifically induced by PAA condition.

We further asked if any specific miRNAs regulate MMP expression. As shown in Fig. 9, PAA induced AKT expression compared with the cells on PS. That prompted us to investigate if PAA induced other gene expression. The difference between PS and PAA was PAA; a soft native environment (1-kPa) allows tumor cells to be invasive, while on PS, a stiff (100-kPa) surface is with reverse effect. Therefore, we focused on MMP9 and MMP2 expression because they are the enzymes responsible for tumor invasion.

Huang and colleagues found that human miR-373 and miR-520c stimulated cancer cell migration and invasion in vitro and in vivo, and that certain cancer cell lines depend on endogenous miR-373 activity to migrate efficiently [26]. Further clinical specimen analyses show that significant upregulation of miR-373 that correlated inversely with CD44 expression. These implicate that miR-373 and miR-520c promote tumor invasion and metastasis [26]. This is consistent with our data showing that on PAA, a soft native environment (1-kPa) allows tumor cells to be invasive while on PS, a stiff (100-kPa) surface is with reverse effect.

We observed an induction of MMP activity by TNFα and TGFβ2 when they were added to F3Y cells on a standard polystyrene culture plate but a reduction of MMP activity when the cells were cultured on a softer hydrogel plates (Fig. 12A and B). Our study predicted a large-scale increase in microRNAs from PS to the PAA environment. A previous study on fibrosarcoma cells showed an increase in MMP9 activity could be explained by miR-520c and miR-373 that acts by targeting the 3’UTR of mTOR and SIRT1 and its downstream effectors and kinases to inactivating signaling pathways that negatively regulate MMP9 expression [21]. Our study predicted that both mir-520c and miR-373 binding sites were common among the 3’ UTRs of genes downregulated on PAA compared to PS (S1 Dataset). This suggests that these same microRNAs were upregulated on PAA and we choose to confirm this by performing RT-PCR on reported and predicted miR-520c and miR-373 targets that are involved in MMP regulation and TNFα and TGFβ signaling. We also hypothesized that the change in the softer elasticity of PAA caused a change in the cell’s physiology that cause a decrease in MMP actively upon introduction of these cytokines and examine key genes from pathways that may influence MMP expression. This is a component of NF-κB, a transcription factor for MMP9 [20, 27] and the receptor for TGFβ2, TGFβR2, which are both targeted by miR-520c and miR-373 [20]. We also examined CD44, a well-established biomarker for PNET in children[28] and with prognostic value[29], which is involved in the activation of MMP9 [30], and is targeted by miR-520c and miR-373 by suppressing CD44 translation [26,31]. As miR-520c [32] was upregulated on PAA (Fig. 12C), however, CD44 expression was downregulated on PAA (Fig. 12D). In addition, we found that these changes were due to the physical property of matrix, but not due to chemical signals (monomers or PAA degradation products) (Fig. 12D).

## Discussion

PAA plate is a very mature material and has been well studied for the impact of their physical elasticity on cell fate determination [12,33]. PAA produces linear stiffness gradients of at least 115 kPa/mm, extending from one kPa (brain stiffness equivalent) to 240 kPa (bone stiffness equivalent). This work addressed the role of substrate elasticity on gene expression of cancer cells. Specifically, we used whole genome microarrays to measure transcriptional expression and identify processes that are affected by the difference in the elasticity of the surface medium. The preferential downregulation of transcripts was identified when cells were grown on PAA. The silencing of those transcripts may be caused by miRNAs. The presences of transcripts containing intron sequences in PNET cells on PS may be a source of miRNA silencing. A cellular change in the functional response to cytokines was due to the change in tissue elasticity. A signaling pathway (AKT) regulated by tissue elasticity was determined from the microarray data. The cancer cells’ AKT-regulated physiologically relevant signaling was examined by gelatin zymography. This was used to measure cell invasiveness by examining the expression and activity of MMPs that degrade ECM, thereby creating space for cellular growth and invasion. We showed the downregulation of several ECM proteins on PAA. The identification of possible miRNA as biological markers is specific for PNETs and brain cancer.

The elasticity of tissue ECM *in vivo* is determined by a balance between the secretion of ECM proteins and the degradation of the ECM by their proteases for cells. For cells grown on softer tissue, the balance favors more protease secretion, while on stiffer and less elastic tissue; more ECM proteins would be expected. Cancer cells grown on the softer PAA surface may recognize it as a native ECM (brain environment equivalent) and may adapt to the environment. Tissue elasticity has been shown to influence the differentiation of normal stem cells towards specific cellular lineages, showing that substrate elasticity determines stem cell fate [34]. We have discussed the possible advantages and disadvantages of using PS, PAA, agarose, organotypic culture, and animal models [1]. We have shown that stem cells migrate through fiber tracks in organotypic culture [9]. Cancers are thought to originate from CSCs, which like normal stem cells, would be affected by the elasticity of the tissue. Here, we examined how cells of a specific type of brain tumor type, PNET, respond to culture conditions that more closely resemble the elasticity of brain tissue compared to standard culture plate conditions. The growth of cells on the softer elasticity of the PAA surface may have a cellular response and behavior that more closely to *in vivo* environmental conditions. Cells grown on standard PS plates are less representative of normal tissue milieu.

By using cell invasiveness as an index of the simulated conditions for in vivo brain, we first performed the functional response of PNET cells to the soft PAA or rigid PS surfaces by examining the cell invasiveness related MMPs enzymatic activity with gelatin zymography. We further studied if MMPs are regulated by cytokines due to the change in tissue elasticity at the protein level. The difference in gene expression patterns between the PNET cells cultured on PS and PAA attracted us focusing on how miRNA regulation can affect the gene expression between the two tissue culture environments, and investigating if there are physiological changes that occur in cells between the PS and PAA culture conditions.

Our data supports the hypothesis that changes in tissue elasticity of cells can affect gene regulation by miRNAs. Furthermore, novel miRNA products may rise from the alternative polyadenylation of intron sequences containing primary miRNA sequences. Cells grown *in vivo* may also use alternative polyadenylation to regulate miRNA expression and that the set of miRNA expressed would vary in different tissue types.

Our analysis of differentially regulated genes between the PS and PAA culture conditions showed a majority of probes downregulated on PAA (negative fold change) instead of upregulating on PAA (positive fold change) (Figs. 2, 3; S3, S4, S5, S6 Datasets). Unbalanced gene expression is known to be common in cancer [35,36,37], which may reflect the differentiation of the cells away from a normal cell phenotype. A method for analyzing genes given the complexity of differential expression like our sample has not yet been developed and widely used. Common methods assume that gene distribution follows a normal distribution and that differentially expressed genes are expected to be equally distributed between upregulated and downregulated genes, and that only a small amount of genes change between different types of treatments[36].

We identified specific miRNAs that enriched on PAA and may be regulated by the elasticity of the surface type. The increase in numbers of miRNA on targeting genes was recognized as a critical factor for the gene downregulated on PAA. To show that the predicted miRNAs was dependent on the elasticity of the cell, comparison of the number of unique miRNAs per gene was analyzed to show that individual miRNA had higher chance to bind to genes downregulated on PAA rather than binding of a gene upregulated on PAA (Fig. 5). The Wilcoxon ranked sum test showed that miRNA binding was greater in the genes downregulated on PAA than due to chance. The Wilconox ranked sum test was performed instead of a paired t-test because we could not assume a normal distribution from the sampled probes. This probability (with statistically significant p-values) was an estimate of how likely a miRNA binding site would be found on a gene grown on PAA or PS.

The finding of multiple miRNA genes that are part of clusters being enriched in the set of genes downregulated on PAA was an evidence to support our hypothesis. miRNAs within clusters were expressed and regulated together, which in turn to silence a groups of targeting genes. So the clustered expressing miRNAs play an important role in gene expression pattern on different cultural conditions. The expression pattern will be a characteristic of a cell in a given extracellular condition. These clustered expressing miRNA such as the C14MC miRNA cluster (S1 Dataset) can be used as marker molecules for PNET cells cultured on PAA. The identification of miRNAs enriched in the brain specific C14MC miRNA cluster and the C19MC cluster shown us the evidence that PAA is able to mimic neuronal tissue [38,39].

The identification of stem cell specific miRNAs(miR-520, miR-302, miR-372, and miR-373) [40,41], which was predicted to be increased on PAA is an indication that the PAA tissue environment may allow the PNET cells to return to a less differentiated state (S1 Dataset). They were all identified to have down regulated mRNA expression on PAA but because they have identical seed sequences we cannot determine to what degree, each miRNA is upregulated without further study. They may all target the same sites and have similar function to increase cell "stemness”. The other miRNAs that were predicted to vary between PAA and PS (S1 Dataset) may be potential cancer cell, stem cell, and diagnostic markers specific tumor types that have not been characterized as of yet.

Functional enrichment of differentially regulated genes was performed to identify gene ontology (GO) terms and KEGG pathways that may change between the two surface conditions and to help discover why miRNAs may be downregulated on PAA. Many terms and pathways were enriched on PAA (S3 Dataset) but closer examination of the probes involved showed that most of the terms and pathways enriched were caused or highly influenced by probes which were downregulated on PAA. Since the terms and pathways identified to be functionally enriched also show bias from the asymmetrical distribution of the differentiated genes, the identification of the terms and pathways would not be ideal in separating the causes from the effects of miRNA silencing on PAA. Another source of difference in miRNA expression between the cells on PS and PAA come from alternative polyadenylation. About half of all miRNA are within intron regions of coding regions and the other half are within the intron and exon regions of non-coding RNA [42]. Alternative polyadenylation of these sites can create alternative 3' UTR that have different binding sites recognized by a tissue specific miRNA. Alternative polyadenylation that result in a short 3'-UTR would have less targets for miRNAs and a long 3'-UTR would have in more possible targets to be regulated by miRNAs. In our model, alternative polyadenylation of primary-miRNA (pri-miRNA) within introns can act to prevent miRNA from being processed. A single pri-miRNA may contain one to six miRNA precursors (pre-miRNA). If we fit the results into our model, mRNA from PNET cells grown on PS are alternatively polyadenylated within introns of coding sequences, but when the cells are grown on PAA, newly processed miRNA that is induced by the PAA growth conditioned binds to the 3' UTR and rapidly degrades the mRNA rather than a slow decay. The newly processed miRNA is dependent on the polyadenylation site of pri-miRNA. The increase in miRNA predicted when ours cells were grown on PAA suggest that polyadenylation occurs further downstream in pri-miRNA which would result in more miRNA produced. Pri-miRNA can also be downregulated by other miRNA or from their own miRNA that they code for which act as a negative feedback mechanism on the production of their own miRNA and effect the regulation of other mRNA and miRNAs and so on. The opposite may also occur where alternative polyadenylation isoforms are produced that avoid downregulation by miRNA.

**Fig 13:**
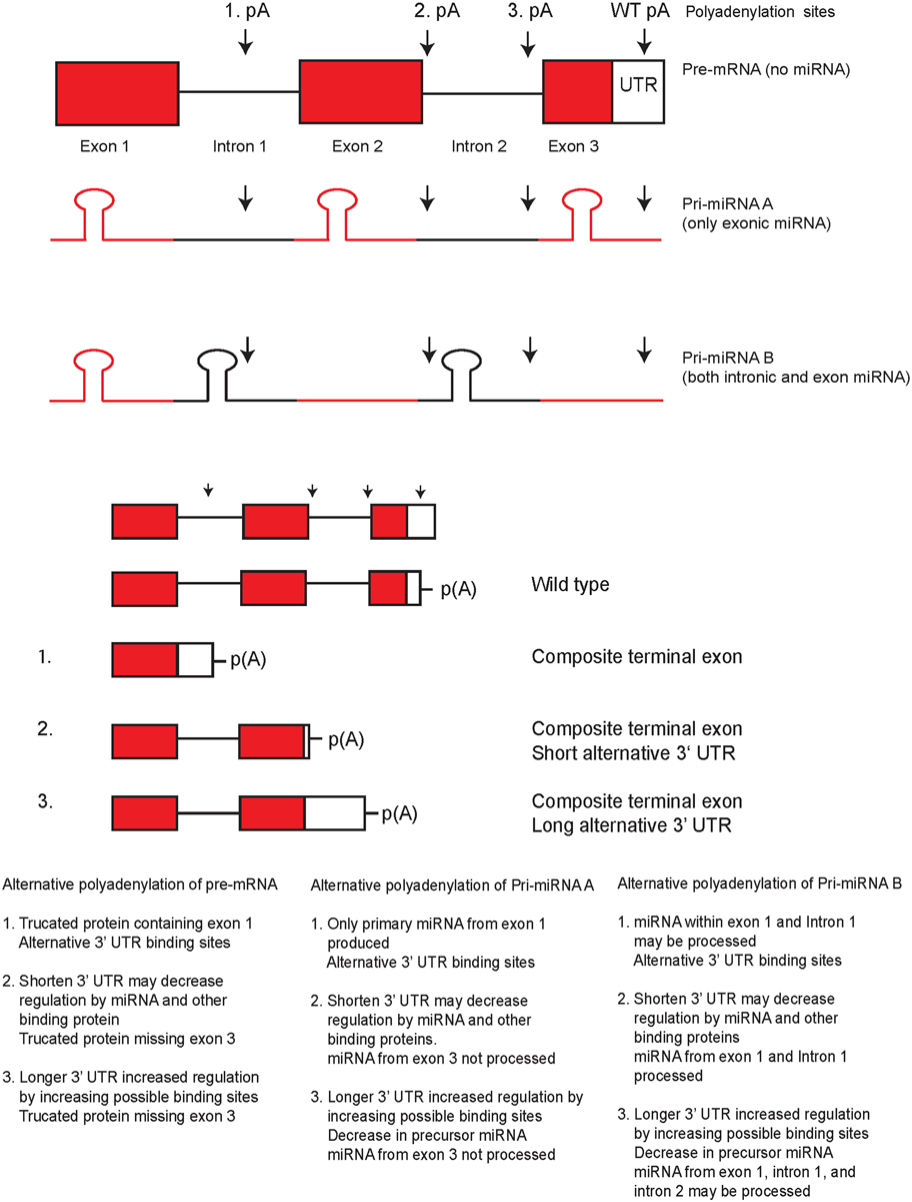
Model of miRNA regulation by alternative polyadenylation sites located within introns. Alternative polyadenylation causes a change in possible miRNA binding sites and truncation of mRNA. Polyadenylation that results in a shortening 3'-UTR generally lowers regulation by miRNA, while a lengthening 3'-UTR increases regulation by miRNA. Alternative polyadenylation of pri-miRNA affects the ability of miRNA to be produced by preventing miRNA stem loop regions from being processed. Alternative polyadenylation sites within the 3' UTR o f a transcripts can have a shorter or longer 3' UTRs. Alternative polyadenylation within introns sequences creates a transcript with an extended coding region that end at the first stop codon within an intron and also creates a 3' UTR. The alternative polyadenylation within an exon does not create a 3'-UTR region.

To elaborate how alternative polyadenylation sites on PS are downgraded on PAA, we show that on PAA, alternative polyadenylation of introns can induce a new set of miRNA not present on PS (Fig. 13). Some alternative polyadenylation sites that were originally present on PS would be silenced by the newly synthesized miRNA. Synthesis of new miRNA also cause miRNA levels to rise and increase binding to 3' UTR sites of Dicer and to the newly synthesized pri-miRNA which would also be silenced if it had the same target sites on its 3' UTR. The negative feedback of Dicer would prevent the continued production of miRNA that are not negatively regulated by specific miRNA and over time miRNA not produced are degraded and not replaced and result in a change in the characteristics of the cell.

The softer PAA surface represents a cellular microenvironment that may be closer to native cell conditions, while the PS condition represents a microenvironment that is closer to that of stiffer tissue. The negative feedback mechanisms that control miRNA and Dicer may also control the expression of genes on different types of tissue. Cells grown on bone, muscle, and brain tissue may have a set of tissue specific miRNA that is induced in their microenvironment and regulated by Dicer and miRNA negative feedback mechanisms.

The process of the metastasis of tumors, the migration of cancer cells and stem cells from one tissue type to another, and the ability of the cell to adapt to its new environment would be dependent on the ability of the cell to recognize its ECM environment and adapt to it to survive, grow, and replicate. This study suggests that the mechanism that allows cells to adapt to their environment involves miRNA regulation of gene expression which is influenced by the elasticity of surface environment. When the cells do adapt to the environment, the behavior of the cell and the response to external stimulus is also changed.

MicroRNA 520c and 373 (miR-520c and miR-373) have been characterized as oncogenes in prostate cancer [31]), cancer cell metastasis-promoting factors in breast cancer [26], and tumor suppressors in estrogen receptor negative breast cancer [20]. Both miR-520c and miR-373 confer robustness to biological processes by upregulation of activity of MMP9 in human fibrosarcoma cells [21]. Mechanistically, both miR-520c and miR-373 upregulate MMP9 expression by targeting mTOR and SIRT1-mediated Ras/Raf/MEK/Erk signaling pathway and NF-κB factor. Our results suggest a change in the physiology of the PNET cells when they are grown on PAA based on gelatin zymography of the PNET cells and their responses to cytokines. Other laboratories have found such changes in MMP activity responding to cytokines in different cell types. These include skin [22], decidual cells of the placenta [43], nucleus pulposus tissue of intervertebral disc [44], and MMP2 of glioma [45]; however, it has not been documented on reduced MMP2 or MMP9 activity, reversed in response to the cytokines signal. In this study, a large proportion of genes were differentially regulated between the PS and PAA culture environments that include ECM and cell receptor genes that receive and transmit cytokine signals as well as many downstream genes from those signals.

The MMPs changes on PS could be interpreted as a decrease in cell invasiveness. However, when the cells are grown on PAA, the induction of MMP activity by cytokines would be interpreted as increasing cell invasiveness. This indicates that experiments performed on cells grown on standard PS cultures plates may not reflect *in vivo* conditions and that tissue elasticity can affect a cell's physiology such that responses to cancer drugs and treatments may not have the desired effect if studies were only conducted on standard PS culture plates. Nevertheless, the expression of miR-520c and miR373 involved in the up regulation of MMP9 could nicely explain the experiments described on the modulation of cytokines activity by the substrate stiffness on MMP activities. This involvement of miRNA expression will be further directly measured by managing miR-520c and miR373 expression in the brain-like microenvironment. The extent of downregulation of mRNA on PAA would suggest that the miRNA negative feedback mechanism acts like a biological switch, which can rapidly turn on miRNA but limits it to a certain level of expression. But because miRNAs have a reported half-life of ~5 days [46], switching the miRNAs off may take several weeks and remains to be investigated. This study only tracks the expression of the cell over a few days and further studies would be needed to observe the effect of long-term growth on PAA compared to PS. We will further confirm the data obtained from PAA matrix by using an *ex vivo* organotypic brain slice system and in vivo animal models.

## Materials and Methods

### PAA Hydrogel Preparation

Hydrogels were prepared as described previously [33,47] with some modifications. A 5% acrylamide and 0.1% bis-acrylamide solution (1.25 mL of 40% acrylamide (Bio-Rad), 0.50 mL of bis-acrylamide (Bio-Rad), 0.100 mL of 1M HEPES, and 8.15 mL of dH2O) was poured that had a resulting expected modulus of elasticity of ~3.15 kPa ± 0.85 for use with cells of neurological tissue origin. To catalyze the reaction, 50 μl of ammonium persulfate and 5μl of TEMED were added to the solution. The solution was passed through a syringe filter and poured over a glass plate with 0.5 mm spacers and a second glass plate was placed over the solution to sandwich the liquid between two glass plates. The solution was allowed to gel under the fume hood for 30 minutes. The top plate was carefully removed and a gel punch was used to cut circular gels that are transferred onto the wells of a six well plate. A 0.5 mM solution of sulfo-SANPAH (Pierce) in 50 mM HEPES pH 8.5 and 0.25% DMSO was prepared and used to cover the surface of the gel. The gel surface was exposed to UV light (365 nm) at a distance of 2.5 inches for 10 minutes to covalently bind the sulfo-SANPAH onto the gel. The wells were washed two times with 50 mM HEPES and addition sulfo-SANPAH solution was added and exposed to UV light for an additional 10 minutes. The wells were then rinsed three times with the 50 mM HEPES solution. A solution of 1μg/mL Collagen type I (BD Biosciences) in PBS was added to the wells to coat the surface of the gel by attaching to sulfo-SANPAH and was refrigerated overnight. The gels were then washed twice with PBS. Cells were then seeded onto the hydrogel.

### Cell Culturing and Harvesting

The cells used in the microarray study were derived from neurospheres in neural stem cells election medium isolated from a three year old female patient with a PNET (F3Y) as approved by our institutional review board [10]. The cells were seeded on six welled tissue culture polystyrene plates coated with collagen type I or on six well tissue plates containing a 5% polyacrylamide gel coated with collagen type I. The cells were grown in and maintained in a solution of 5% FBS in Advanced D-MEM (Gibco). Cells were harvested by aliquoting both the cells and the medium into 15 mL conical tubes. The tubes were then centrifuged at 1600 rpm for 5 minutes. The cell pellet was used for RNA extraction and Western blot analysis while and the supernatant (conditional medium) was used for Zymography.

### RNA Extraction and Analysis

To extract total RNA, the cell pellet was treated with Trizol reagent (Invitrogen) according to the manufacturer’s instructions. RNA concentration and purity was measured with a spectrophotometer at 260/280 nm. Total RNA was stored at-80°C. In experiments where cytokines were added to culture, the cells were serum starved in 0.1% FBS plus Advanced DMEM overnight before cytokines were added and then maintained in 5% FBS-Advanced DMEM. The cells and supernatant were harvested three days afterwards.

Affymetrix GeneChip U133 Plus 2.0 arrays was used to profile the gene expression change of F3Ycells grown on two different surface mediums. Cells was plated either on a standard polystyrene collagen I coated culture plate or on a 5% polyacrylamide collagen I coated Petri dish. The cells were lysed with Trizol after three days and the RNA was then extracted and quantified. RNA was processed and hybridized onto six arrays (three replicates from the PS sample and three from the PAA sample). Labeling, hybridization, and scanning of the arrays were performed at USC core faculty.

### Microarray Chip Data Analysis

The resulting intensity files (CEL files) were analyses with dChip software [48] using the following parameters to compute the signal values and normalized the data set: model-based expression model, mismatch probe (PM/MM difference) background selection, and invariant set or quantile normalization. In order to filter and identify relevant genes only probes that had a signal intensity value of over 100 in greater than 50% of the arrays was used to account for possible systematic errors such as background signal noise and variations in individual gene chips. Differentially regulated genes were identified by examining the fold change (FC, mean experimental signal (PAA)/mean control signal (PS), if FC < 1, the negative inverse of the value was reported) between the probes signals of the two samples conditions. Probes with a |FC| > 2were used to generate a list of probes corresponding to differentially regulated genes for further analysis. All determination of FC was calculated using the lower 90% confidence bound of FC and a p-value for paired t-test lower than 0.05. Similar list was generated for varying fold changes to examine sets of higher degree of differentiation.

To determine if miRNA was a factor in the negative regulation of genes when grown on PAA, a randomly selected sample size of 50 highly downregulated genes (FC < -10) was compared to 50 upregulated genes (FC > 2) to obtain statistically significant results. The Entrez Gene ID of these samples was used to search for and collect predicted miRNA binding sites. Targets sites were searched using the August 2010 release of microRNA.org, which is a resource to identify miRNA binding sites. It utilizes a miRNA target prediction algorithm, miRanda, and scores by mirSVR to search for target genes that may be regulated by a miRNA and also for miRNA that may be targeting the 3' UTR of a particular gene. Only conserved miRNA binding sites with a good mirSVR score (≤ -0.10) were kept. If more than one 3' UTR was listed, only the first listed was included in the analysis. Predicted miRNA and its genes targets were tabulated for each set and annotated with its mature sequences, accession number, and chromosomal location. The Wilcoxon ranked sum test was then used to determine if predicted miRNA binding sites were greater on genes downregulated on PAA than on PS.

A method to identify significant miRNAs based on expression arrays was performed using Diana-MirExTra by comparing miRNA predicted from differentially regulated genes to miRNA predicted for a set of unregulated genes as previously described [49,50,51]. Affymetrix probe IDs were converted into Ensembl Gene IDs needed as an input for use with Diana-MirExTra using the Database for Annotation, Visualization and Integrated Discovery (DAVID) gene ID conversion tool [52,53]. Only one Ensembl Gene ID was kept in situations where Affymetrix Probe Ids have multiple possible Ensembl Gene IDs. Diana-MirExtra was used to show that the predicted miRNAs of a set of differentially regulated genes was significantly greater than the predicted miRNAs from a list of genes defined as unregulated. In my analyses, I chose DIANA-microT v3.0 to predict miRNAs. A list of miRNAs and its significance (p-value) that the difference between the differentially regulated and unregulated gene sets is due to chance was created (S2 Dataset).

Diana-MirExtra was used to show the probability that the set of differentially regulated genes had a higher miTG score from the list of background genes for individual miRNAs. Higher miTG score are more likely to correctly predict target sites. The list of miRNAs with its associated significance (p-value) and number of genes over the DIANA-microT threshold score was collected and analyzed (S2 Dataset).

Gene enrichment analysis was performed by using DAVID v6.7 to compare the differentially regulated genes to annotated gene ontologies, pathways, and databases to search for biological functions that are enriched from the differentially regulated genes [52]. The differentially regulated list of probes (|FC| > 2) was compared to background list composed from all the probes with a signal intensity of more than 100 for more than 50% ofthe probes. Relevant enriched pathways were mapped onto KEGG biological pathways [54].

Ensembl program was used to overlay microarray probes onto individual gene sequences [55]. An option to display HGU133 Plus 2.0 probes on the gene sequence was enabled that allowed the determination if the regions where the probes reside was upregulated or downregulated or if the probe was complimentary to a intron region or if it spanned an exon region. This determination was made by importing and comparing NCBI human RefSeq transcripts [56] to the microarray probe tracks which were both aligned to a gene of interest on the Ensembl genomic sequence viewer. Probes that was mapped to the gene in question but not specific to the location being analyzed was discarded from further analyses. Probes that did not align to human RefSeq transcripts were considered to be complimentary to intron regions.

### Bradford Protein Assay

The protein content in conditional medium and cell lysates was quantified using Bio-Rad Protein Assay solution (Bio-Rad) [57]. One part of the solution was mixed with four parts dH_2_O. 200 μl of the solution was added to each wells of a 96 well plate and 10 μl of the sample were added and mixed and incubated for 5 minutes. Bovine serum albumin was dissolved in dH_2_O to generate a standard curve over arrange of 0.2–1.0 mg/mL protein. Absorbance was measured at 595nm using a micro plate reader (Molecular Devices).

### Zymography

Gelatin zymography was performed using a 10% SDS-PAGE containing 20mg/mL gelatin (Sigma) in dH_2_O. Samples were equalized with the same amount of protein from cell lysate or the conditional medium were prepared and loaded into the wells of the SDS-PAGE. Purified MMP-9 was used as a MMP9 positive control and 3T3 condition medium used as an MMP2 positive control. The gels were run at a 100 V until finished. Gels were rinsed for 30 minutes in 2.5%TritonX-100, washed with dH_2_O and incubated overnight in developmental buffer (2 mM CaCl_2_, 120 mM NaCl, 50 mM Tris-Cl pH 7.5) at 37°C. Gels were stained with 0.5% Coomassie Blue R-250 for 1 hour and destained in destaining buffer (30% methanol, 10% acetic acid, 60% ddH_2_0) for 1 hour and imaged. Bands of clearing indicate enzymatic degradation of the gelatin substrate.

### Sepharose Bead Pull-down Assay

Gelatin Sepharose beads 4B (71–7094–00, GE Healthcare) was used to concentrate MMP proteins for use with gelatin zymography [58]. Thirty-microliter gelatin-conjugated Sepharose 4B bead suspension was transferred into fresh Eppendorf tube and centrifuged at 1,000 rpm and the supernatant was discarded. The beads were washed with 500 μl of a buffer solution (50 mM Tris-Cl pH 7.5, 150 mM NaCl) and centrifuged for two minutes at 1,000 rpm. The supernatant was discarded and then 1.5 mL of conditioned medium was added. The tube was rocked for an hour at 4°C and then centrifuged for two minutes and the supernatant discarded. The beads were then washed with 500 μl of the buffer solution, centrifuged for two minutes, and the supernatant discarded. The previous step was then repeated leaving the beads. 20 μl of zymogram buffer (Bio-Rad) was added to elute the MMPs proteins and the sample was loaded into the gelatin zymogram gel wells.

### RT-PCR

All RT-PCR conditions were described previously [10]. Primers were designed as follows: hsa-mir-520c pri-miRNA [32] (Forward: 63.4 ^o^C (5p-fw)—accgctctctagagggaagcac and reverse: 63.5 ^o^C (lv-R)—agaagcccaccatcatccatc) with products of 1364bp and CD44 (Forward constitutive (58.2 ^o^C—catcccagacgaagacagtc and reverse constitutive-tttgctccaccttcttgactcc) with products of 1299bp. The data was analyzed by following the procedures [59].

### Western Blotting

To prepare samples for western blotting, the cell pellet was washed with 1x PBS (Hyclone) and centrifuged. The supernatant was discarded and the cell pellet then lysed with TBS-T (20 mM Tris pH 7.6, 150 mM NaCl, 1% Triton X-100, with phosphatase inhibitors (10 μg/mL Aprotinin, 10 μg/mL Leupeptin, 5 μg/mL Pepstatin, 1 mM PMSF, 1 mM NaF, 1 mM Na_3_VO_4_) added [60]. A 10% SDS-PAGE gel was prepared for western blotting. Equal amount of protein taken from the cell lysates were loaded into the wells of the gel. Samples were then heated for 10 minutes at 90°C, loaded into the wells, and the gels run at 100 V. The gel was transferred onto a PVDF membrane at 220 mA overnight and the membrane then blocked with 5% dry milk (Carnation) and 1% BSA in TBST solution for 1 hour. Primary antibody (1:200) was added to the solution and left overnight at 4°C. The antibody used were AKT1/2/3 (sc-8312, Santa Cruz Biotech), p-AKT (sc-16646-R, Santa Cruz Biotech), Actin (sc-1615, Santa Cruz Biotech), MMP2 (sc-6838, Santa Cruz Biotech), and GAPDH (Santa Cruz Biotech). The membrane was washed three times with TBST and a secondary HRP-conjugated antibody was then added (1:10000) and incubated for two hours. The PVDF membrane was again washed three times with TBST. A chemiluminescence solution (Amersham) was added to the membrane, and the protein complexes were visualized using a chemiluminescent film [61].

## Supporting Information

S1 Dataset Identification of miRNA candidates that are differentially regulated by tissue elasticity. (XLS)

S2 Dataset Comparison of up and downregulated gene sets to a set of genes unchanged by tissue elasticity. (XLS)

S3 Dataset GO biological process terms enriched by genes differentially regulated between PS and PAA. (XLS)

S4 Dataset GO cellular component terms enriched by differentially regulated probes. Probes differentially regulated (FC > |2|) were compared to a background gene expression to determine enriched terms using DAVID v6.7 software.
(XLS)

S5 Dataset GO molecular function terms enriched by genes differentially regulated between PS and PAA. (XLS)

S6 Dataset KEGG pathways enriched by genes differentially regulated between PS and PAA. (XLS)
