# Correction: Tissue Elasticity Regulated Tumor Gene Expression: Implication for Diagnostic Biomarkers of Primitive Neuroectodermal Tumor

**DOI:** 10.1371/journal.pone.0128504

**Published:** 2015-05-19

**Authors:** Long T. Vu, Vic Keschrumrus, Xi Zhang, Jiang F. Zhong, Qingning Su, Mustafa H. Kabeer, William G. Loudon, Shengwen Calvin Li

There are errors in the Funding Section. The correct Funding is as follows: This study was supported by the Children's Hospital of Orange County (CHOC) Children's Foundation, CHOC Neuroscience Institute, Austin Ford Tribute Fund, W. M. Keck Foundation, Grants R01CA164509 (National Institutes of Health), CHE-1213161 (National Science Foundation), and a Seed Grant from University of Southern California. The funders had no role in study design, data collection and analysis, decision to publish, or preparation of the manuscript.

Affiliation #6 is incorrect. The affiliation should be: Department of Perio, Diagnostic Sciences, Biochemistry, Ostrow School of Dentistry, and Department of Pediatrics, Keck School of Medicine, University of Southern California, Los Angeles, CA 90089, United States of America.

There is an error in affiliation for the third author. Xi Zhang is affiliated with #6 and with #10 Department of Hematology, Xinqiao Hospital, Third Military Medical University, 400037, Chongqing, China.
